# Transcriptome analyses reveal protein and domain families that delineate stage-related development in the economically important parasitic nematodes, *Ostertagia ostertagi* and *Cooperia oncophora*

**DOI:** 10.1186/1471-2164-14-118

**Published:** 2013-02-22

**Authors:** Esley Heizer, Dante S Zarlenga, Bruce Rosa, Xin Gao, Robin B Gasser, Jessie De Graef, Peter Geldhof, Makedonka Mitreva

**Affiliations:** 1 The Genome Institute, Washington University School of Medicine, St. Louis, Missouri 63108, USA; 2 U.S. Department of Agriculture, Agricultural Research Service, Animal Parasitic Diseases Lab, Beltsville, Maryland 20705, USA; 3 Department of Veterinary Science, The University of Melbourne, 3030, Werribee, VIC, Australia; 4 Department of Virology, Parasitology and Immunology, Faculty of Veterinary Medicine, Ghent University, Merelbeke 9820, Belgium; 5 Department of Medicine, Division of Infectious Diseases, Washington University School of Medicine, St. Louis 63110, Missouri, USA; 6 Department of Genetics, Washington University School of Medicine, 63108, St. Louis, Missouri, USA

**Keywords:** Cattle, Parasite, Nematode, Transcripts, *Ostertagia ostertagi*, *Cooperia oncophora*, Comparative genomics

## Abstract

**Background:**

*Cooperia oncophora* and *Ostertagia ostertagi* are among the most important gastrointestinal nematodes of cattle worldwide. The economic losses caused by these parasites are on the order of hundreds of millions of dollars per year. Conventional treatment of these parasites is through anthelmintic drugs; however, as resistance to anthelmintics increases, overall effectiveness has begun decreasing. New methods of control and alternative drug targets are necessary. In-depth analysis of transcriptomic data can help provide these targets.

**Results:**

The assembly of 8.7 million and 11 million sequences from *C. oncophora* and *O. ostertagi*, respectively, resulted in 29,900 and 34,792 transcripts. Among these, 69% and 73% of the predicted peptides encoded by *C. oncophora* and *O. ostertagi* had homologues in other nematodes. Approximately 21% and 24% were constitutively expressed in both species, respectively; however, the numbers of transcripts that were stage specific were much smaller (~1% of the transcripts expressed in a stage). Approximately 21% of the transcripts in *C. oncophora* and 22% in *O. ostertagi* were up-regulated in a particular stage. Functional molecular signatures were detected for 46% and 35% of the transcripts in *C. oncophora* and *O. ostertagi*, respectively. More in-depth examinations of the most prevalent domains led to knowledge of gene expression changes between the free-living (egg, L1, L2 and L3 sheathed) and parasitic (L3 exsheathed, L4, and adult) stages. Domains previously implicated in growth and development such as chromo domains and the MADF domain tended to dominate in the free-living stages. In contrast, domains potentially involved in feeding such as the zinc finger and CAP domains dominated in the parasitic stages. Pathway analyses showed significant associations between life-cycle stages and peptides involved in energy metabolism in *O. ostertagi* whereas metabolism of cofactors and vitamins were specifically up-regulated in the parasitic stages of *C. oncophora.* Substantial differences were observed also between Gene Ontology terms associated with free-living and parasitic stages.

**Conclusions:**

This study characterized transcriptomes from multiple life stages from both *C. oncophora* and *O. ostertagi.* These data represent an important resource for studying these parasites. The results of this study show distinct differences in the genes involved in the free-living and parasitic life cycle stages. The data produced will enable better annotation of the upcoming genome sequences and will allow future comparative analyses of the biology, evolution and adaptation to parasitism in nematodes.

## Background

Studies on the free-living nematode *Caenorhabditis elegans* have provided a wealth of information on metazoan biology and development. However, being a member of the Nematoda has periodically engendered erroneous assumptions that *C. elegans* is a measurable representative of other nematodes within this phylum. More recent studies on the genomes and transcriptomes of other nematodes have demonstrated the extensive diversity within this group and the need to look more closely at individual genera to begin addressing questions related to nematode parasitism and host-parasite relationships.

*Cooperia oncophora* and *Ostertagia ostertagi* are two parasitic nematodes of the order Strongylida that belong to the same phylogenetic clade as *C. elegans*[1]. Both species are parasites of bovids in more temperate regions of the world. The diseases caused by these nematodes are among the most costly to the cattle industry where hundreds of millions of dollars are lost each year in lower productivity and higher management expenses. Treatment of cattle infected with these strongylid nematodes commonly involves anthelmintic drugs; however, similar to what has been observed in many microorganisms, drug resistance has become a significant problem within this group of parasites [2]. In spite of their economic impact, a dearth of information is available on their molecular biology.

Parasites of the genera *Cooperia* and *Ostertagia* as well as other Strongylida exhibit similar life cycles that begin with fertilized eggs being passed in the host feces. Like *C. elegans*, the first three larval stages (L1, L2, and L3) are considered free-living because they are environmentally-exposed but with no host dependency. The infective L3 has a protective sheath (L3sh) that allows for movement on pasture while protecting the parasite from ecological pressures. Upon ingestion, however, the nematodes become host-dependent i.e. parasitic; the L3 exsheath (L3ex), develop to the fourth larval stage (L4) and continue development to adults in the abomasum (*Ostertagia*) or the intestines (*Cooperia*). Despite their biological similarities, infection by *O. ostertagi* does not confer strong immunity against reinfection except in cattle which have been infected for extended periods of time [3]. Cattle which have been infected by *C. oncophora*, on the other hand, attain resistant to reinfection more readily [4]. Furthermore, even though cattle are often found simultaneously-infected with both species, anthelmintic resistance has only been documented in *Cooperia* spp.

Deciphering the underlying biological differences between these two similar organisms may open the path for more holistic hypotheses on host-parasite relationships, host immunity, and the development of drug resistance. Detailed and comparative explorations of their transcriptomes and genomes would not only provide insights into fundamental biological processes, but underpin the discovery of new treatments and control methods that may be broadly applicable to other less similar nematodes. Although limited transcriptomic information is available for two developmental stages of *O. ostertagi*, [5] this falls woefully short of representing the entire life cycle and providing insights into what differentiates the free-living and parasitic stages. Currently, no transcriptomic data are publicly available for *C. oncophora*. Analysis of transcriptome data and their comparison with genomic data is well known to provide useful information about an organism [5-7]. This approach has led to studies on identifying new drug targets (e.g. [8-10]), understanding nematode biology [11], and detecting parasite protein-specific indels and evaluating their importance in parasitism and evolution [12], to name a few.

The present study has generated extensive, stage-related information on the transcriptomes of *C. oncophora* and *O. ostertagi*. The comprehensive comparative transcriptomic analysis of stages representing the entire life cycles of these animals established gene expression patterns which characterize and delineate among each of the stages investigated. In addition, transcripts which are unique to free-living or parasitic stages have also been discovered. The resources and results in this study provided molecular insights that improve our understanding of parasite biology and development, and identified differential transcripts among stages/sexes. In broader terms, these analyses will be extremely useful for annotating their upcoming genomes [13] and could enable the development of new methods to control infections by these parasites.

## Results

### Transcript reconstruction and homologs in other nematodes

Sequencing of the transcriptomes of *C. oncophora* and *O. ostertagi* resulted in 9,603,581 and 11,900,750 reads and 29,900 and 34,792 assembled transcripts and corresponding peptide translations, respectively (Table 1). These transcripts represent an estimated 81% and 74% of the complete transcriptomes (defined by detection of the conserved low copy eukaryotic genes [14]) wherein 202 and 184 CEGs were detected in these two species, respectively. The transcript consensus sequences are available at http://nematode.net[15]. The number of transcripts likely over estimates gene discovery, as one gene could be represented by multiple non-overlapping transcript fragments. Such ‘fragmentation’ [16], was estimated at 21% for *C. oncophora* and 22% for *O. ostertagia*.

**Table 1:** Summary of generated reads, assembled trascripts and annotation information

	***C. oncophora***	***O. ostertagi***
Total number of 454 Reads	9603581	11900750
Number of Reads Removed	859727	821428
Number of Reads after Contamination Screening	8743854	11079322
Number of Reads after Clustering	3713617	7079583
Number of Mapped Reads	6588676	8102342
CEG’s	202	184
Number of Predicted Peptides	29900	34792
Number of peptides with InterPro match	13812	12274
Number of peptides with Pfam match	12311	14317
Number of peptides with GO match	10511	16130

**Figure 1:**
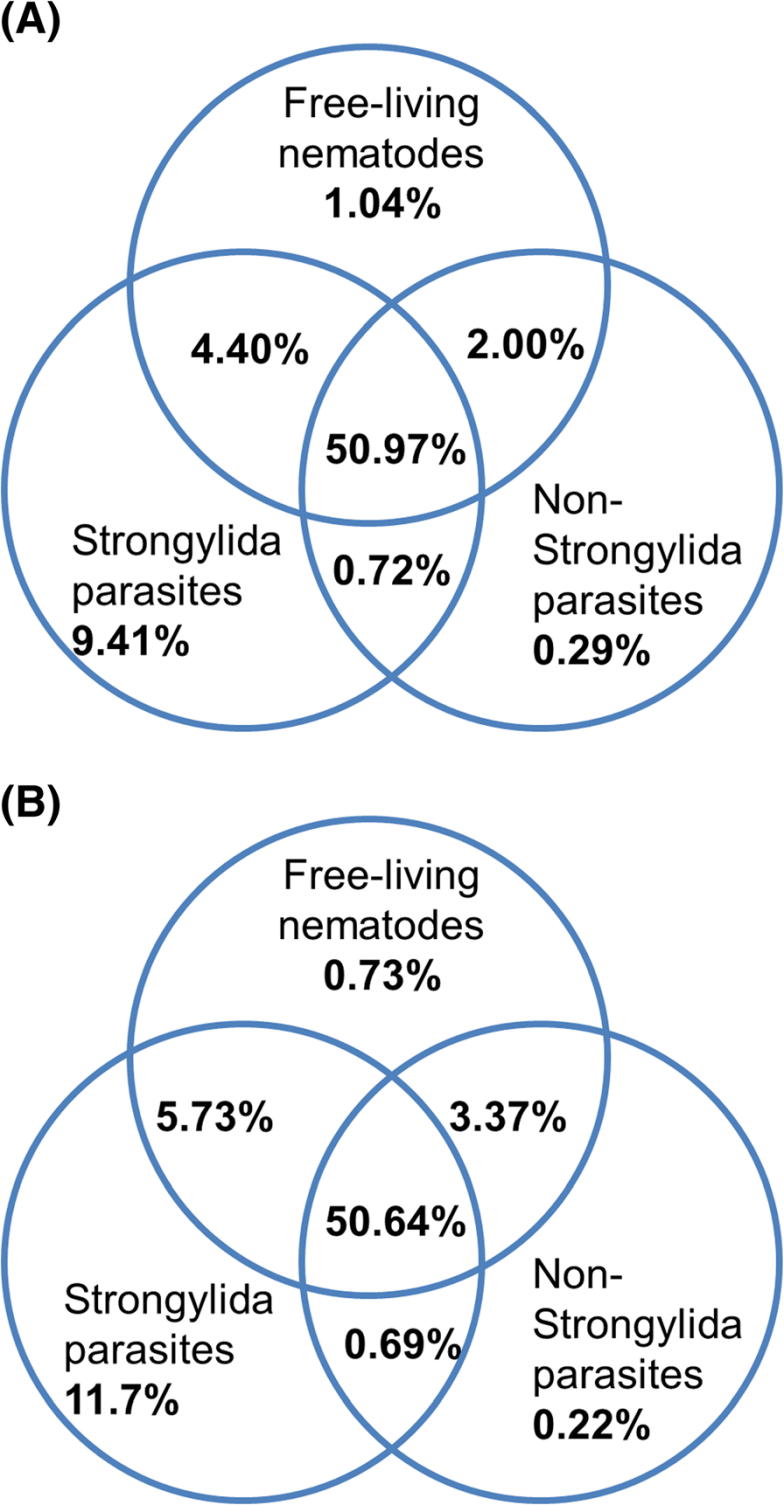
**Distribution of protein homologues in free-living nematodes, Strongylida parasites, and Non-Strongylida parasites.** The percent of homologues in each of the three databases as well as the overlap between the databases is shown. (**A**). *C. oncophora;* (**B**). *O. ostertagi.* For species included in each of the three databases please *see* the Materials and Methods.

Sequence homologues for 68% of the predicted peptides of *C. oncophora* and 73% of those of *O. ostertagi* were found in at least one other nematode species (Figure 1). Approximately half of these homologues were common to sequences in all nematodes examined (*see* Materials and Methods). Strongylids had the largest subset of group specific homologues, while non-strongylid parasite species had the fewest (Additional file 1). Peptides predicted to be species-specific were significantly shorter in length, on average, than peptides with matches in other species (Additional file 2: Figure S1). This explains in part, the perceived sequence specificity in lieu of finding homologs as reported previously [17].

### Transcript profiles throughout the *C. oncophora* and *O. ostertagi* life cycle stages

**Figure 2:**
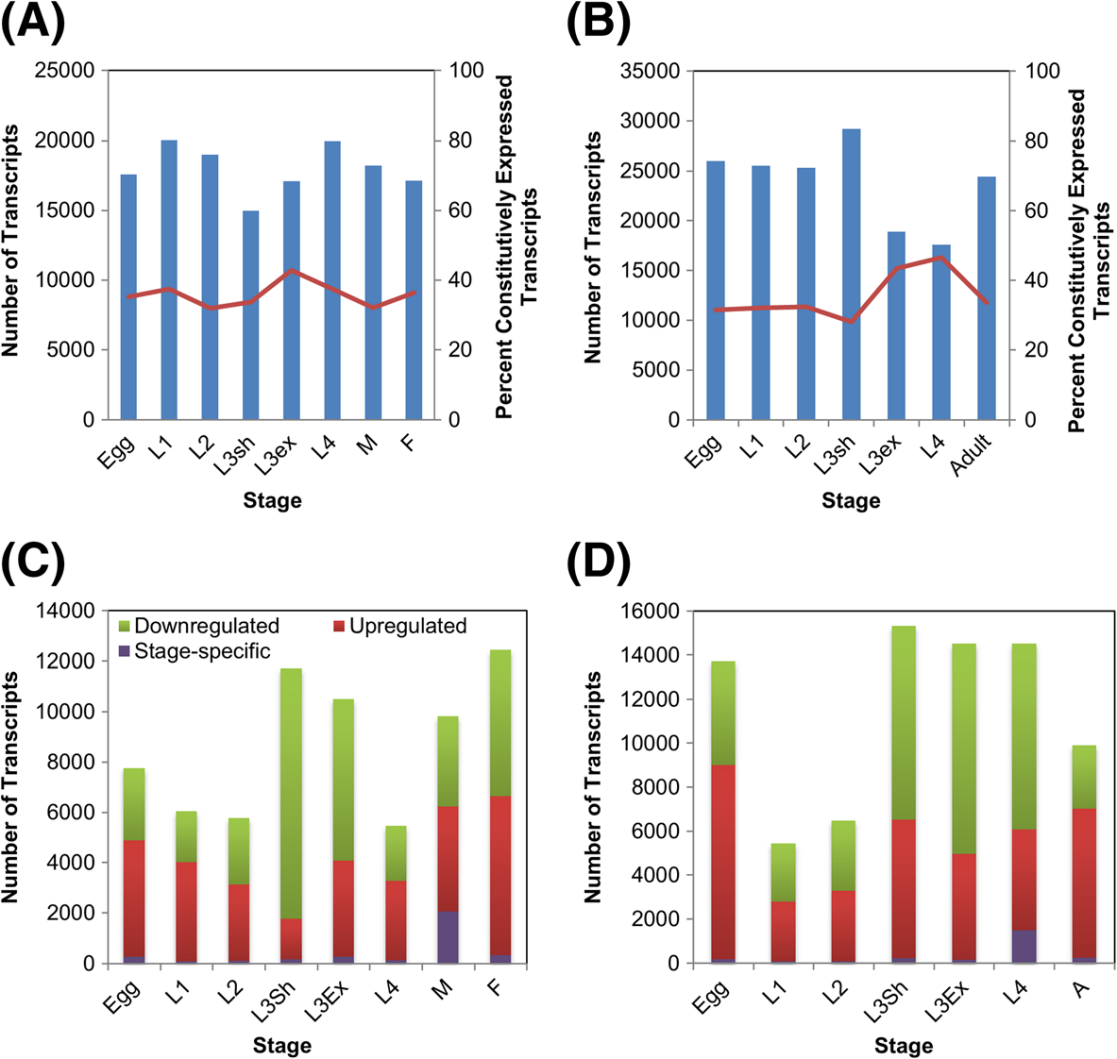
**Transcript expression in different developmental stages.** This figure represents the number of transcripts expressed in each stage and the percent of those transcripts that are constitutively-expressed in *C. oncophora* (**A**) and *O. ostertagi* (**B**). (**C**) *C. oncophora* and (**D**) *O. ostertagi* depict the number of transcripts up-regulated, down-regulated and specific to a given stage.

On average, 35% of the transcripts of a given stage are constitutively expressed in that specie, and this was true for both species (Figure 2A, 2B and Additional file 3). In *C. oncophora,* 21% are found in all stages, whereas 24% are found in all stages of *O. ostertagi*. The KEGG pathways analysis suggests that the majority of these transcripts are involved in genetic information processing and in particular, transcription and translation (Additional files 4 and 5); the InterPro (IPR) domains encoded by these transcripts confirm their associations with these functions (Additional file 6 and 7). One of the most prevalent domains in constitutively-expressed transcripts in both species is ubiquitin-associated/translation elongation factor (IPR015940).

While some of the peptides encoded by constitutively-expressed transcripts may not contain identifiable domains, most of them exhibit homology with other proteins. The majority of these peptides (88% and 90%, respectively) had homologs in at least one specie from the three phylogenetic databases to which they were compared, whereas 79% and 75% have homologs in all three databases suggesting that constitutively-expressed transcripts are involved in core cellular processes. As expected, peptides in *C. oncophora* and *O. ostertagi* had higher numbers of homologs among the Strongylida parasites (~88% in both species) than any other group; the fewest number were shared with the non-Strongylida nematodes.

The number of transcripts expressed in only one stage was small (<1%; Figure 2C, 2D and Additional file 8). In general, transcripts expressed in the later stages i.e. adult, had a high number of homologs (~64%) in other species, whereas those expressed in the earlier stages i.e. egg, had fewer (25% and 17%, respectively). The parasitic stages including the L3sh exhibited a higher number of homologs in the strongylid parasites than in the other two groups of species, whereas more of the transcripts expressed in the free-living stages showed similarity with organisms in the two non-Strongylida groups than with those in the Strongylida group with the exception of the L3sh.

Comparing stage-specific transcript expression within species revealed that the majority of transcripts expressed in each stage are not differentially-expressed (Additional file 8); ~20% of transcripts in both species are up-regulated in any given stage whereas ~26% are down-regulated. Comparative values for up- and down-regulated transcripts are shown in Figure 2C and 2D. On average 74% and 68% of up-regulated transcripts have homologs in at least one nematode group to which they were compared; up-regulated transcripts had a higher number of homologs to Strongylida parasites only (70% for *C. oncophora* and 73% for *O. ostertagi)*. As with the constitutively-expressed transcripts, translation is the most prevalent KEGG category in both *C. oncophora* and *O. ostertagi*. Most transcripts (~89% in *C. oncophora* and ~93% in *O. ostertagi*) are up-regulated in more than one stage likely resulting from carryover between consecutive stages.

There was a total of 1393 transcripts identified as encoding putatively-secreted peptides of which 538 were enriched in at least one stage. It was determined that free-living stages tended to have more of these transcripts in common with each other than with the parasitic stages. Parasitic stages tended to have a common pool of secreted peptides as well. The exception to this was *C. oncophora* L4 which shared more secreted peptides with the free-living stages than with the other parasitic stages. The 5% of domains most prevalent in the secreted peptides were very similar between the two species. Transthyretin-like, metridin-like ShK toxin, saposin B, and CAP domains were among the most prevalent for secreted proteins in both species. Two insulin domains were among the most prevalent in secreted peptides of *C. oncophora* but were absent from *O. ostertagi*. Ves allergen was found in 16 secreted peptides of *O. ostertagi* but was found in only one secreted peptide of *C. oncophora*.

### Differences in gene expression and associated functions between free-living and parasitic stages

Pfam domains were identified in 41% of the peptides in both *C. oncophora* and *O. ostertagi* matching 2507 and 2658 different domains, respectively. In both organisms the most prevalent domain was RNA recognition motif (PF00076) (Table 2).

**Table 2:** The three most abundant Pfam, InterPro, and GO terms associated with the peptides

		**Number of peptides**	
	**Description**	***C. oncophora***	***O. ostertagi***
Pfam Code			
PF00076	RNA recognition motif	208	226
PF00069	Protein kinase domain	170	192
PF01060	Transthyretin-like family	164	169
IPR code			
IPR016040	NAD(P) - binding domain	269	336
IPR011009	Protein kinase-like domain	252	271
IPR012677	Nucleotide-binding alpha-beta plait	244	284
GO code			
Biological process			
GO:0006508	oxidation-reduction process	759	989
GO:0008152	metabolic process	713	842
GO:0006457	proteolysis	492	579
Cellular component			
GO:0005622	intracellular	745	769
GO:0016020	membrane	575	729
GO:0016021	integral to membrane	544	626
Molecular Function			
GO:0005515	protein binding	1537	1735
GO:0005524	ATP binding	877	1050
GO:0003824	Catalytic activity	848	869

An examination of transcripts expressed in the free-living (egg, L1, L2, L3sh) and parasitic (L3ex, L4, adult) stages of development revealed that some Pfam domains are abundant in both phases of development while others are unique to a single stage or phase. The most abundant Pfam domain (chromo domain) in the free-living stages of *C. oncophora* was expressed solely in this phase of development while two of the top three domains (Lectin C-type domain and trypsin) in the parasitic stages were not expressed in any of the free-living stages (Additional files 9 and 10). Domains like the RNA recognition motif were found equally in both phases.

**Figure 3:**
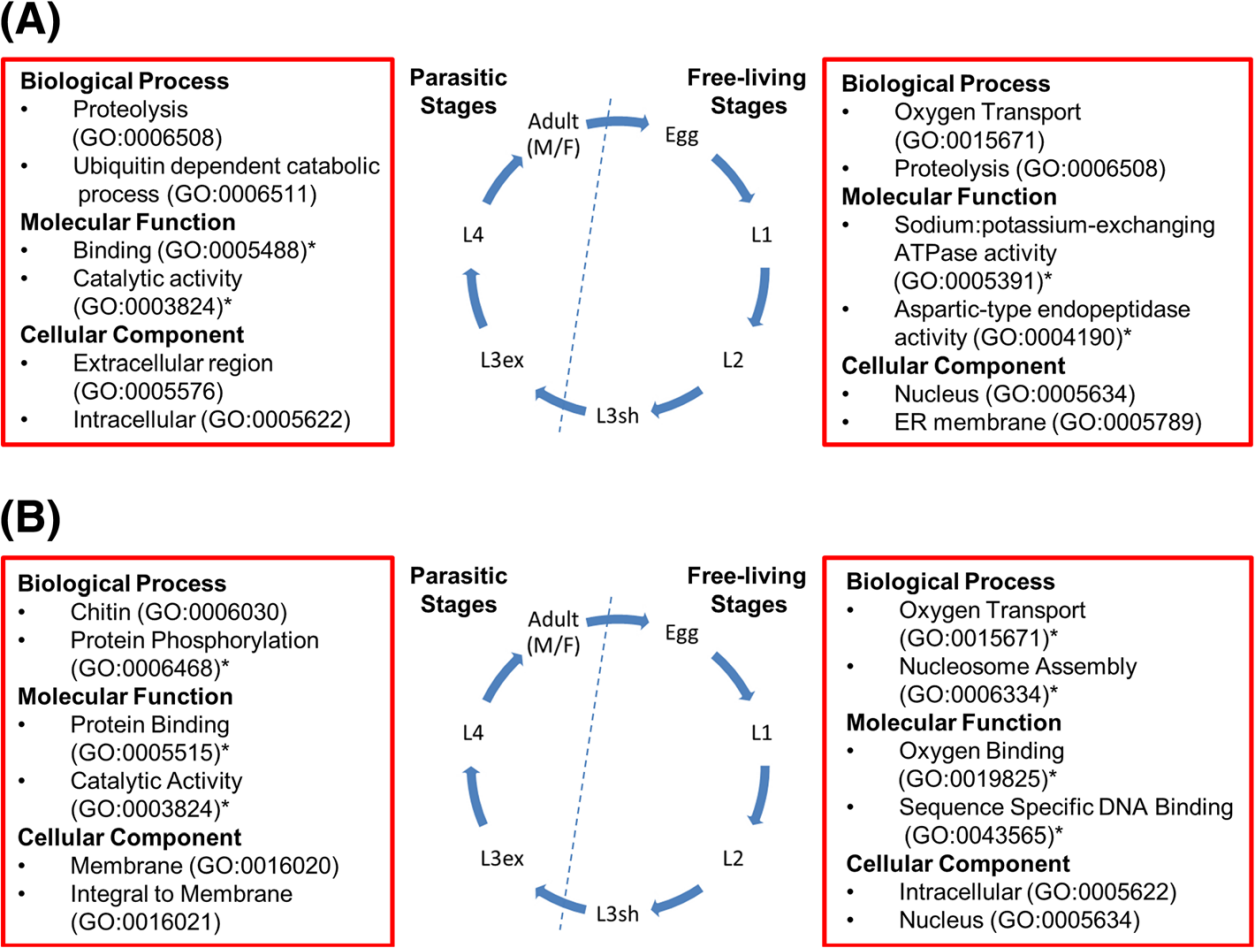
**GO term associations with transcripts expressed in each stage.** For each phase of the life cycle (free-living or parasitic) several prevalent GO terms are listed. * indicates a given term is significantly-enriched in that life cycle stage (*p* < 0.05). (**A**) *C. oncophora*; (**B**) *O. ostertagi*.

A total of 35% of *C. oncophora* peptides and *O. ostertagi* peptides could be associated with GO terms categorized as ‘biological process’, ‘cellular component’, and/or ‘molecular function’ (Table 1 and Additional files 11, 12, 13, 14, 15 and 16). Examination of GO terms associated with the peptides reveals significant differences between parasitic and free-living stages (Figure 3). Significantly-enriched molecular functions in the parasitic stages of *O. ostertagi* and *C. oncophora* included binding (GO:0005488), protein binding (GO:0005515), and catalytic activity (GO:0003824). In the free-living stages, sodium:potassium-exchanging ATPase activity (GO:0005391) and aspartic-type endopeptidase activity (GO:0004190) were enriched in *C. oncophora* while oxygen binding (GO:0019825) and sequence specific DNA binding (GO:0043565) were enriched in *O. ostertagi*.

**Figure 4:**
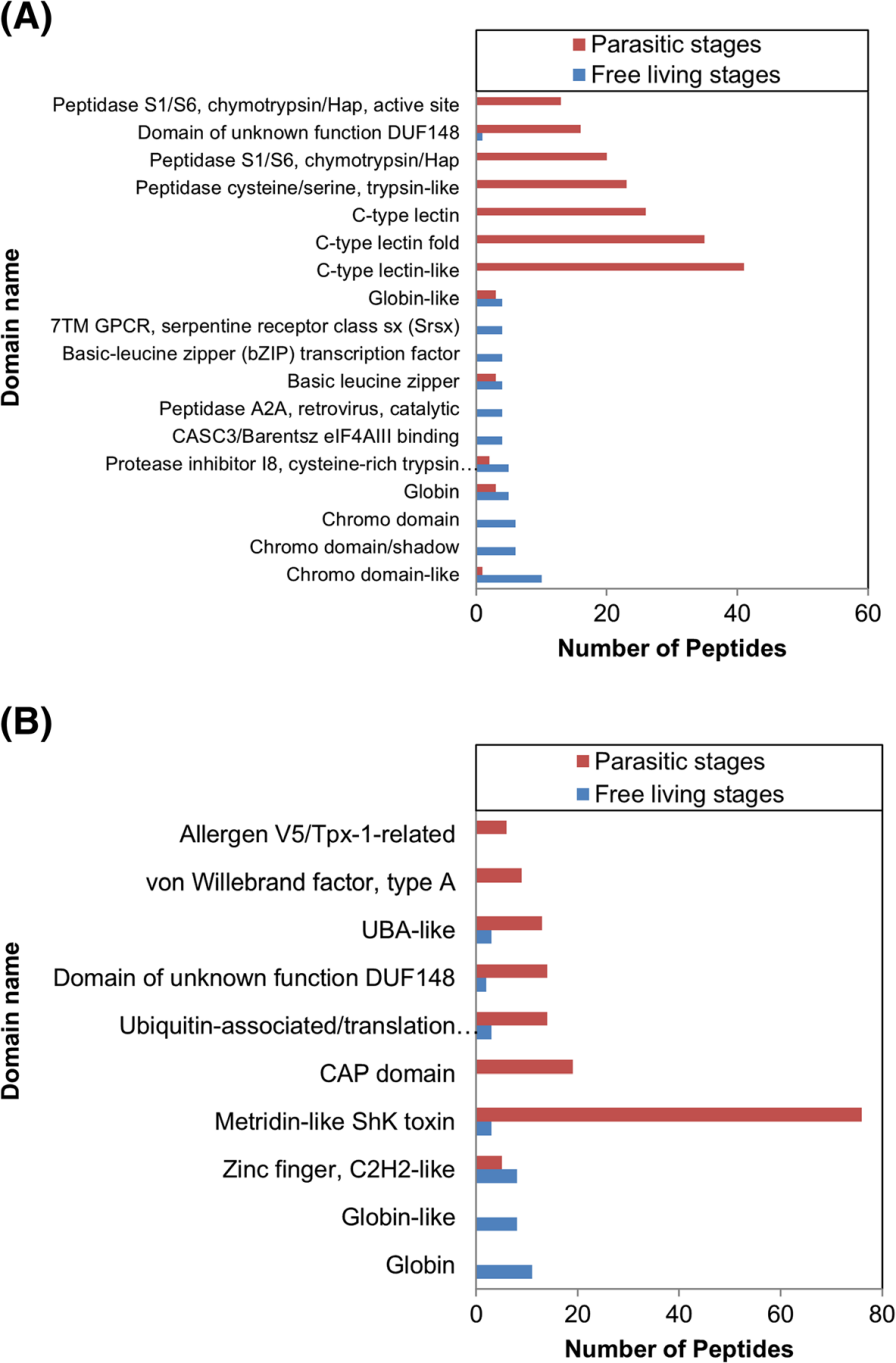
**Significantly-enriched (*p*** **< 0.05) InterPro domains in the free-living (egg, L1, L2, L3sh) or parasitic (L3ex, L4 and adult) stages of (A) *C. oncophora *and (B) *O. ostertagi*.**

**Figure 5:**
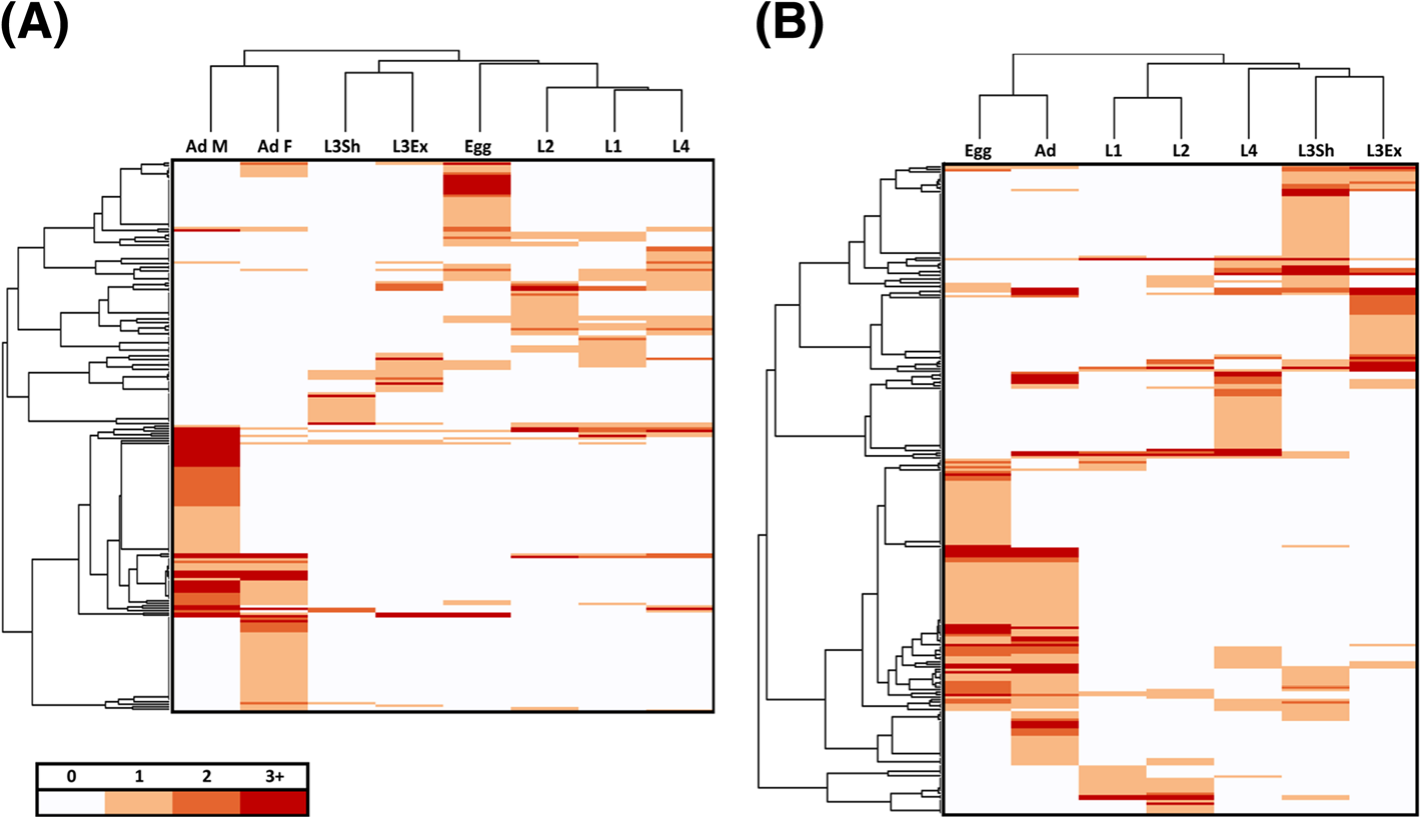
**Clustering of stages based upon the number of transcripts in a stage containing a specific InterPro domain.** (**A**) *C. oncophora*; (**B**) *O.ostertagi.* A lower-range scale (0 to 3+) was used to better illustrate the similarities and differences between the stages.

A total of 4,160 and 4,135 unique InterPro domains were detected in 46% of *C. oncophora* and 35.3% of *O. ostertagi* peptides with the most prevalent domain being ‘NAD (P)-binding’ domain (Table 2). In the free-living stages, globin, zinc finger domains, and chromo domains were among the most prevalent (Figure 4 and Additional files 17 and 18). In the parasitic stages, metridin-like ShK toxin, CAP domain, and C-type lectins were among the most prevalent motifs (Figure 4). Clustering based on the number of IPR domains found in up-regulated peptides revealed that consecutive stages tend mainly to cluster together with the exception of peptides from the egg (Figure 5). In both species, the domains found in these peptides tend to be linked to the adult stage, which is likely due to the presence of fertilized eggs in the adults.

**Figure 6:**
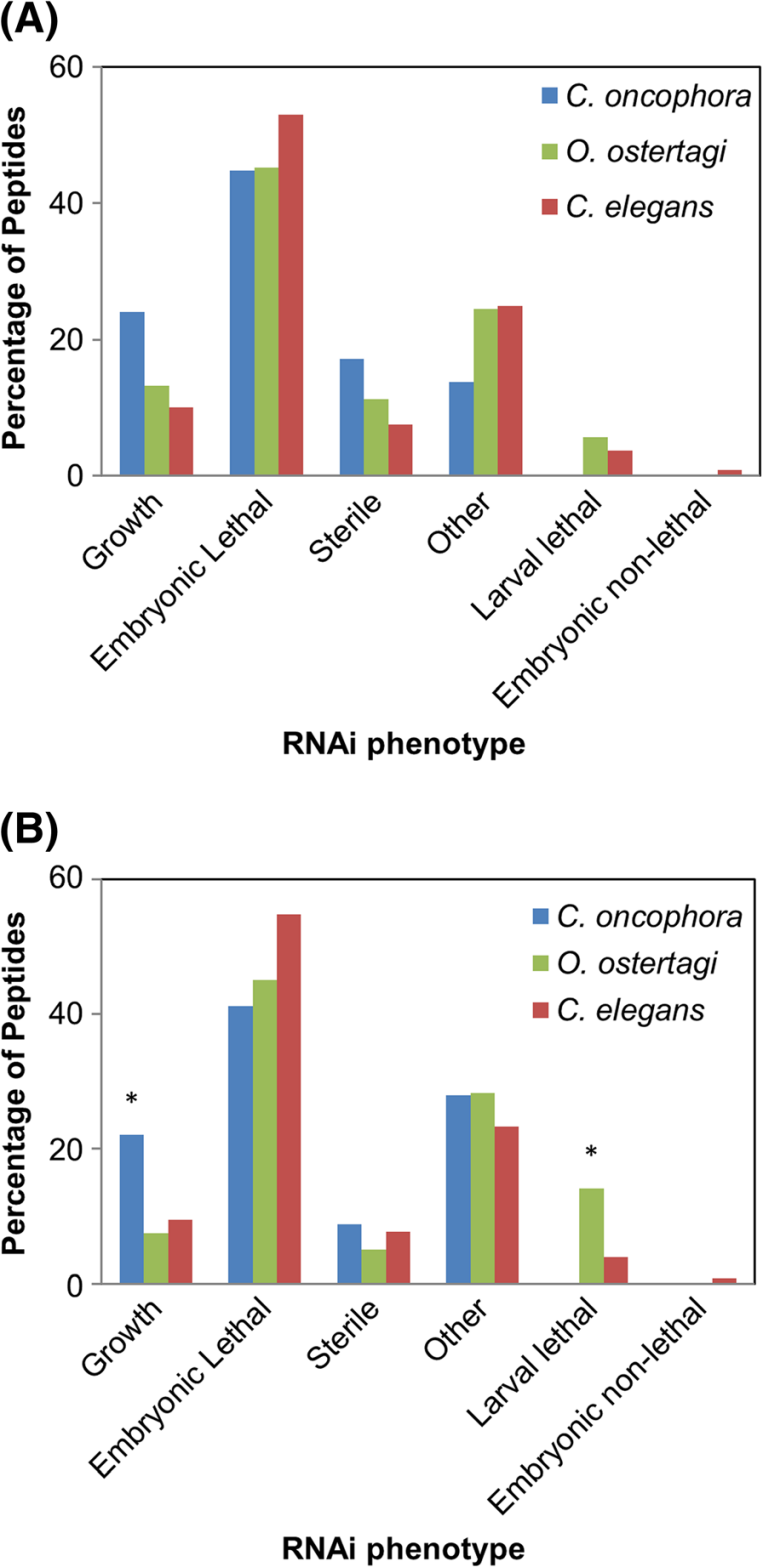
**Comparison of phenotype distribution between RNAi-surveyed *C. elegans *genes, and *C. onchophora *and *O. ostertagi *homologues to *C. elegans *genes with similar phenotypes.** (**A**). The percent of *C. oncophora* peptides encoded by transcripts expressed in free-living stages (egg, L1, L2 and L3sh) with homologs to *C. elegans* proteins with various RNAi phenotypes. (**B**). The percent of *O. ostertagi* peptides encoded by transcripts expressed in parasitic stages (L3ex, L4 and adult) with homologs to *C. elegans* proteins with various RNAi phenotypes. “*” indicates that for a specific RNAi phenotype, significantly more (*p* < 0.05) peptides in either *C. oncophora* or *O. ostertagi* exhibited that phenotype than did *C. elegans*.

*C. elegans* had 8,896 proteins with RNAi phenotypes in the stages analogous to free-living *C. oncophora* and *O. ostertagi,* and 8,205 proteins in the parasitic stages (i.e. post dauer). *C. oncophora* had 29 polypeptides from the free-living stages and 68 from the parasitic stages with homologs to the *C. elegans* genes with available RNAi phenotypes, whereas *O. ostertagi* shared 53 homologous polypeptides from free-living stages and 120 polypeptides from the parasitic stages, with *C. elegans* genes of known RNAi phenotype. For most RNAi phenotypes inferred, there were no significant differences between the numbers of polypeptides in the two species and the numbers of proteins in *C. elegans* that exhibited those phenotypes. *C. oncophora* had significantly more peptides with predicted RNAi growth phenotypes (*p* = .007) in the parasitic stages when compared to *C. elegans*. In contrast, *O. ostertagi* exhibited a significantly greater number of peptides with larval lethal phenotypes (*p* = 2.8E-05) in the parasitic stages (Figure 6) relative to *C. elegans*.

**Figure 7:**
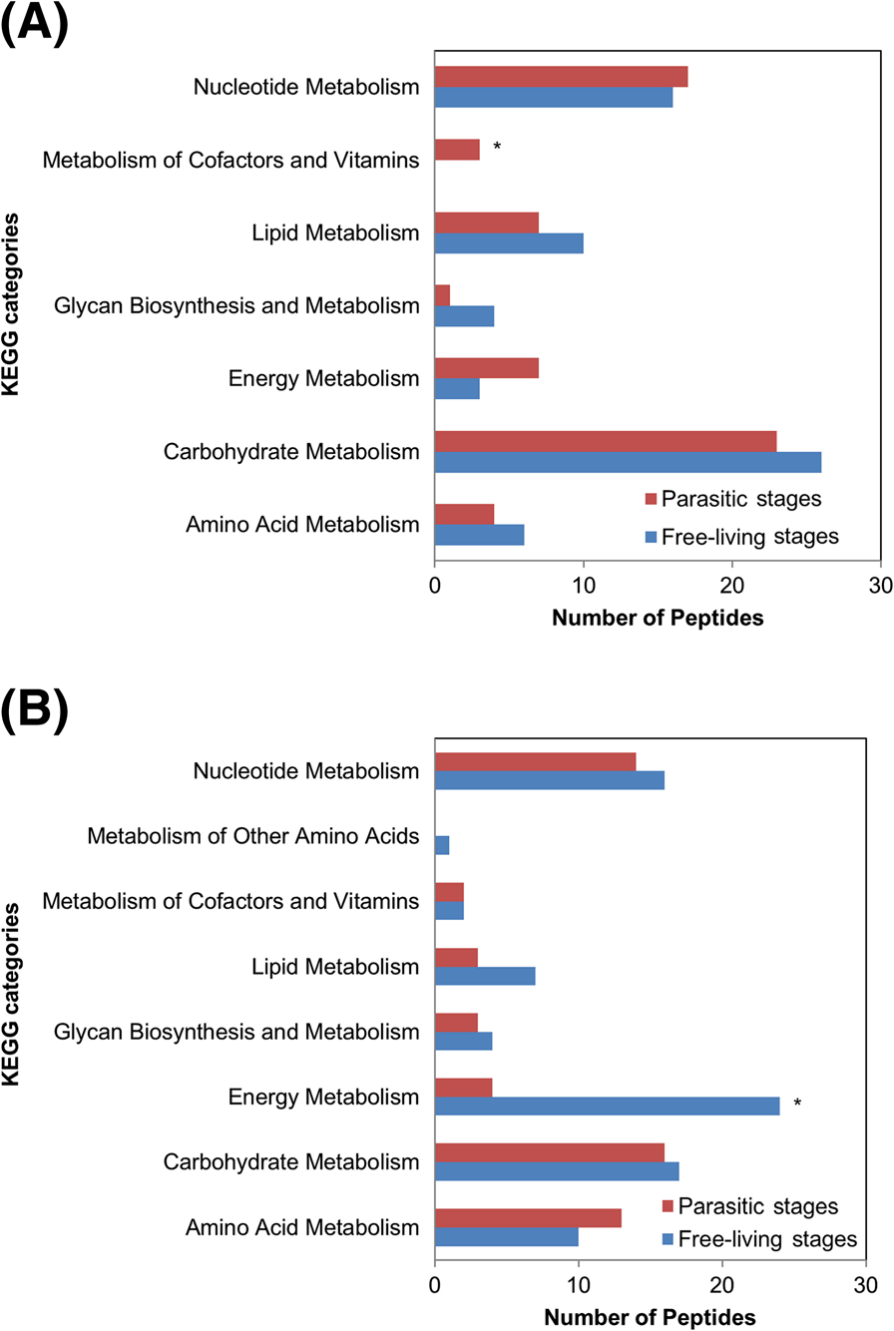
**Distribution of KEGG categories associated with up-regulated transcripts.** The number of up-regulated transcripts in free-living and parasitic life stages associated with KEGG categories is compared in (**A**) *C. oncophora* and (**B**) *O. ostertagi*. * indicates significance (*p* <0.05).

Comparison of the up-regulated transcripts to the KEGG pathways revealed an increase in the number of transcripts involved in metabolism of cofactors and vitamins in the parasitic stages of *C. oncophora* (*p* = 0.04). In the free-living stages of *O. ostertagi,* there were significantly (*p* = 0.01) more transcripts involved in energy metabolism when compared to the parasitic stages (Figure 7).

## Discussion

The gastrointestinal parasites studied here exhibit numerous biological similarities. They begin their lives as eggs that are passed in the feces from the host. They remain as free-living organisms up to and including the L3sh at which time they are ingested by the host, ex-sheath and then continue their development as parasitic organisms within the host. Examination of transcripts in both species revealed that 68.8% in *C. oncophora* and 73.0% in *O. ostertagi* have sequence homologues in the other species examined in this study (Figure 1) and that ~60% of strongylid genes have homologs in *C. elegans*[7,18]. While we have identified few peptides (0.2% and 0.3%, respectively) that share homology only to non-Strongylida species (Additional file 1), mainly *Ascaris. suum* and *Brugia malayi*, these are likely homologous peptides not yet identified in other Stongylida species because of the incomplete nature of their genome sequences. Our study showed similar results in that BLAST searches identified homologous sequences in 55.1% of *C. oncophora* and 57.9% of *O. ostertagi* polypeptides when compared with free-living nematodes. The slightly higher percentages observed in this study can be attributed to the better coverage of the *Cooperia* and *Ostertagia* transcriptomes using pyrosequencing relative to the coverage obtained from conventional EST libraries in previous investigations (e.g. [5]). Because of differences in the environments and living requirements between the free-living and parasitic stages, it is expected that some pathways and enzymes will be unique to these two phases of development and coincide with the requirements and challenges imposed by the different environments. Comparisons of domains and pathways present in the free-living stages to those in the parasitic stages revealed many of these differences.

Given the similarities between *C. oncophora* and *O. ostertagi,* it was not unexpected that there would be significant overlap in the domains found in up-regulated peptides in the various stages. For example, among the 20 most abundant domains in all stages, ten were identical in both organisms. The domains that were prevalent in the free-living vs. parasitic stages may provide clues to the lifestyles and environments in which these organisms live. In the free-living stages, domains previously implicated in growth and development tended to dominate. In *C. oncophora* three different chromo domains (IPR000953, IPR016197, and IPR023780) and the MADF domain (IPR006578) were enriched (Figure 4, Additional file 17). Chromo domains are often found in association with heterochromatin protein 1 (HP1) which functions in germline and vulval development in *C. elegans*[19]. The MADF domain is a transcription factor in *Drosophila* that activates genes necessary for development [20]. Chromo domains and MADF domains were found in proteins that predominate in the egg as would be expected (Additional file 18). Interestingly, the chromo domain (IPR000953) and MADF domain (IPR006578) were also found elevated in adult *O. ostertagi*. Two domains identified as basic leucine zippers [21] (IPR004827 and IPR011700) were up-regulated in the free-living stages of *O. ostertag*i.

As the organisms transition to L1, the domain prevalence shifts as well. In *C. oncophora*, the most prevalent domain was EF-hand-like domain (IPR011992). This domain tends to be found in calcium binding proteins [22]. In contrast, the most prevalent domain in *O. ostertagi* was globin (IPR012292). Globin and saposin domains were prevalent in the L2 of both species. Both of these domains were found in secreted peptides of both species. Saposin domains are expressed in all stages of *Ancylostoma caninum*[23]. While they were not found in enriched peptides in every stage of *C. oncophora* or *O. ostertagi*, these domain containing peptides were expressed in all stages.

During the L3sh, the worms both protect themselves from environmental stress as well as prepare for uptake by and development within the host. Among the most prevalent domains in the L3sh were protease inhibitor I8 (IPR002919) and late embryogenesis abundant (LEA) protein (IPR004238) in *C. oncophora* and *O. ostertagi*, respectively. Among the multitude of roles played by protease inhibitors, it has been suggested that they are also involved in protecting invading organisms from host molecules, in particular, those derived from the gastrointestinal tract, such as pepsin. In this way gastrointestinal nematodes can safely navigate and survive within the host digestive tract [6]. Late embryogenesis abundant proteins have been shown to play a role in protection from the environment. In *Aphelenchus avenae* (plant pathogenic roundworm), LEA proteins help protect other proteins from aggregating during times of low water and possibly play a role in preventing desiccation [24].

During the parasitic stages beginning with the L3ex, it is expected that transcriptional profiles will shift towards host interaction while maintaining those profiles associated with worm development. Zinc finger domains (IPR007087 and IPR015880) which are important in cell differentiation and development [25] were indeed among the most prevalent domains in the L3ex of *C. oncophora* and in *O. ostertagi* adults possibly resulting from additional rapid growth as the worms emerge from the gastric glands. In *O. ostertagi* L3ex, the most prevalent domains found in the greatest number of peptides, were DUF148 and metridin-like ShK toxin. The metridin-like ShK toxin domain (IPR003582) was up-regulated in *O. ostertagi* parasitic stages and was the most prevalent domain in the L4 stage. Noteworthy is that the metridin-like ShK toxin domain is often found near the C-terminus of *C. elegans* metallopeptidases. It is suggested that these domains are important in parasitic interactions [26]. CAP domains (IPR014044) were also among the most prevalent domains in *C. oncophora* L4 and *O. ostertagi* adults (Figure 4 and Additional files 17 and 18); however, among putatively-secreted peptides, CAP domains were observed in *C. oncophora* L3sh, L4, and adults, and in *O. ostertagi* L4. In mammalian species, proteins harboring CAP domains are divided into nine subfamilies which encompass cysteine-rich secretory proteins (CRISPs). Similar CRISP domains (PF00188) were up-regulated in *Ostertagia* (Additional file 10) and have recently been identified in the *Lethenteron japonicum* (parasitic lamprey) [27] which secretes a CRISP containing protein from its buccal glands once it has attached to the host. It is believed that this CRISP protein enhances vasodilation and feeding [28]. It should be noted that the concept of “secretory proteins” is defined as a cellular event and not necessarily a function related to parasites secretions. As such, there need not be a direct relationship between CRISP proteins and “extraorganismal” functionality i.e., parasite secretory products. Case in point, in mammals, CRISP proteins are well known to be associated with cell signaling, reproduction, fertilization and the maturation of spermatozoa. As such, it may not be coincidental that in parasites, an abundance of CRISP proteins is associated with the later larval and adult stages of worm development. CRISP domains have been found associated with proteins with immunomodulatory activity [29] and have been studied in few parasitic nematode species including the hookworm *A. caninum*[30] and the murine strongylid nematode, *Heligmosomoides polygyrus*[31].

It is well known that proteins such as chymotrypsin, trypsin, and peptidase [32] are involved in the breakdown of proteins into constituent parts. Chymotrypsin (IPR001254) domains were up-regulated in the parasitic stages of *C. oncophora* and found only in the parasitic stages of *O. ostertagi* (Figure 4 and Additional file 17 and 18); trypsin-like domains (IPR009003) were up-regulated in *C. oncophora*, and; peptidase S1/S6 (IPR001254) was one of the most prevalent domains in female *C. oncophora* (Figure 4 and Additional file 17). Given their abundance in the later stages of development (e.g. L4 and adult), it is possible that proteins associated with these domains collectively play a role in the feeding process [24]. This is supported in part by the observation that these domains are present in nine secreted peptides in *C. oncophora* and 75 in *O. ostertagi.* It is possible that a subset of these is not only secreted from the cell but also from the parasite. Given that the adult diets of these parasites vary based upon either abomasal (*Ostertagia*) or intestinal (*Cooperia*) contents, these secreted proteases may also participate either in countering the host immune responses (L4) by hydrolyzing antibodies, or in establishment in the host (L3 ex) particularly as it relates to *Ostertagia* and its need to enter the gastric glands and keep inflammation at bay.

The three C-type lectin domains (IPR001304, IPR016186, and IPR016187) were the most prevalent domains in male *C. oncophora* (Additional file 17) and were up-regulated as well in *O. ostertagi* (Additional file 18). As expected, all three of these domains are found in putatively secreted peptides in both species predominantly because evolutionarily, the superfamily of proteins containing C-type lectin domains is comprised of extracellular metazoan proteins with diverse functions. In general, these domains are involved in calcium dependent carbohydrate binding. However, it should also be noted that not all proteins containing C-type lectin domains can actually bind carbohydrates or even Ca^2+^. Indeed, most of the proteins containing this domain and referred to as C-type lectins are not lectins. Nonetheless, those with functionality have been implicated in innate immune responses in invertebrates, and have been linked to proteins involved at the host-parasite interface which may assist in evading the host immune response [33]. As such, differences in the levels of these domains between *C. oncophora* and *O. ostertagi* may in part be associated with the observed variation in host immunity as well as distinction in the predilection sites of the respective L4s and adult worms. A closer investigation of sequence similarity to C-type lectins from free-living and parasitic nematodes and an analysis of the locus to which these proteins are eventually translocated might shed light on physiological functionalities as they relate either to sustaining life within the organism or controlling the host-pathogen interface. Some nematode C-type lectins have been linked to the parasite surface i.e., the epicuticle. Among other things, the nematode cuticle is comprised of collagen proteins and these proteins exhibit stage specific expression [16,34].

Examination of KEGG categories demonstrated significant associations between life cycle stages and peptides involved in energy metabolism in *O. ostertagi* where 24 peptides were found in the free-living stages and only four in the parasitic stages (Figure 7). Further analysis of these 24 polypeptides provided clues about environmental adaptation. From the egg stage through L2, the worms are present in the fecal pat. Upon developing to L3sh they become more motile and migrate from the pat to better position themselves for ingestion by the host. Of the 24 peptides involved in energy metabolism in the free-living stages of development, 17 are associated with methane metabolism. As the free-living stages of both species are found in the fecal pat and the fecal pat is a methane rich environment, this is not surprising. Only one of the 24 peptides is up-regulated in the L3sh and classified as an enzyme involved in oxidative phosphorylation rather than methane metabolism. It is possible that this becomes more functional as the worm distances itself from the fecal pat and readies itself for ingestion by the host. It is also interesting to speculate that environmental queues i.e. host GI tract, may down-regulate transcriptional activity of the proteins involved in methane metabolism and in turn induce exsheathment and worm development.

In *C. oncophora,* the KEGG category “metabolism of cofactors and vitamins” was significantly more abundant in the parasitic stages than in the free-living stages (Figure 7). The specific enzymes involved are associated with pantothenate and CoA biosynthesis [35], and thiamine metabolism. All three peptides were up-regulated only in adult females. Inasmuch as these enzymes were not observed in abundance in fecal eggs, their functions are likely related specifically to females or to egg development *in utero*.

While many of the transcripts were stage specific, others were expressed in all stages. These constitutively-expressed transcripts are likely involved in core molecular processes used to sustain life, as shown by the domains found within them. This conclusion is also bolstered by the embryonic lethal phenotypes predicted for the majority of the constitutively-expressed transcripts that link to an RNAi phenotype in *C. elegans*. These transcripts and their encoded proteins should make attractive drug targets provided sufficient variation can be found between parasite and host proteins.

## Conclusions

Control of parasitic nematodes is routinely accomplished through anthelminthic drugs. Resistance to these drugs is increasingly becoming a problem especially in livestock hosts. To date, resistance has surfaced to nearly all commercially available drugs [36]. In an effort to better understand this resistance and help combat the higher production costs associated with the lack of efficacy, a detailed study of these parasites at a molecular level was conducted. To this end, we have generated comprehensive data on the transcriptomes of all discernible life cycle stages of these two organisms. The genome sequences for *C. oncophora* and *O. ostertagi* have been initiated in an effort to complement and complete this work (http://www.genome.gov/10002154) [13]. The cDNA sequences generated in this study will enable better annotation of these genomes upon completion. In the current study, many differences were revealed between the free-living and parasitic stages of these nematodes when examined at the domain, process and pathway levels. During the free-living stages of development, peptides and pathways involved in growth and development were more prominent. In contrast, peptides, domains and pathways that traditionally function in the degradation of proteins were more prevalent during the parasitic stages. These differences are likely associated with host adaptation and therefore parasitism. Further in-depth examination of the differences in domain prevalence and expression between the free-living and parasitic stages may reveal conservation in genes linked to infection, host recognition, immune response and disease. Equally important is understanding the similarities between evolutionarily related organisms in the hope of detecting biological and molecular threads that link the parasitic stages. In this way, we may better identify targets for the development of new classes of nematocides. Holistic approaches such as this could extend new treatments to human pathogens as well.

## Methods

### Sample preparation, library construction, and sequencing

*Ostertagia ostertagi* eggs were purified from the feces of calves infected with *O. ostertagi* by sequentially sieving diluted fecal material over 400, 150 and 64 μm sieves, and finally collecting the eggs on a 37 μm sieve. To collect L1, the eggs were incubated for 24 h at 23°C in tap water after which the larvae were purified by baermannization. The L2 were collected by culturing the feces for 5 days at 23°C followed by baermannization. The larvae were confirmed to be L2 by measuring them under the microscope. The L3 sheathed (L3sh), L3 exsheathed (L3ex) and L4 were prepared as previously described [37].

Adult parasites of *O. ostertagi* were microscopically-selected from abomasal contents from animals killed 28 days post infection. *Cooperia oncophora* eggs, L1, L2, L3sh and L3ex were also collected as described above. The L4 were obtained by baermannization of intestinal contents and washings from animals euthanized 10 days post infection; adult worms were microscopically collected from animals euthanized 21 days post infection and further partitioned into male [M] and female [F] worms.

Total RNA was prepared by homogenizing all parasite samples in Trizol (Invitrogen). All RNA samples were DNAse treated prior to mRNA isolation and sequencing. The integrity and yield of the RNA was verified by the Bioanalyzer 2100 (Agilent Technologies, Cedar Creek, Texas). Total RNA (3 μg) was treated with Ambion Turbo DNase (Ambion/Applied Biosystems, Austin, TX). Approximately 1.4μg male and 2.7 μg female total RNA were used as the templates for cDNA library construction using the Accuscript HF Reverse Transcriptase Kit (Agilent Technologies, Cedar Creek, Texas) and SMART primers (Invitrogen, Carlsbad, CA). PCR cycle optimization was performed to determine the minimum cycle number to amplify full-length cDNA products using the SMART primers and Clontech Advantage-HF 2 polymerase Mix (Clontech/Takara Bio, Mountain View, CA). Amplification was carried out for 30 cycles for the male sample and 27 cycles for the female sample. PCR cycle optimization was performed with normalized cDNA to determine the threshold cycle number using the SMART primers and Clontech Advantage-HF 2 polymerase mix previously mentioned. The determined number of cycles was 14 for both the male and female samples. Finally, 5’ and 3’ adaptor excision was performed by digestion with Mme1. The excised adaptors were removed utilizing AMPure paramagnetic beads (Agencourt, Beckman Coulter Genomics, Beverly, MA). Five micrograms of the cDNA was run on a 0.8% GTG Seakem agarose gel for size selection (Lonza, Basel, Switzerland). Fragments in the 300–800 bp size range where end polished and ligated to 454 Titanium library adaptors utilizing reagents from the Titanium General Library Kit (Roche 454). An AMPure (Agencourt, Beckman Coulter Genomics, Beverly, MA) bead cleanup was performed to remove library adaptor dimers and cDNA fragments less than 300bp in length. The library was immobilized with Strepavidin beads (Library Immobilization Bead and Buffer Kits from 454 Roche) and single stranded with 0.125N Sodium Hydroxide. The single-stranded library was quantitated by a Quant-it single stranded DNA assay using the Qubit (Invitrogen, Carlsbad, CA) and the integrity validated using the Bianalyzer 2100 (Agilent Technologies, Cedar Creek, Texas). The library fragments were immobilized onto DNA capture beads supplied in the 454 Titanium Clonal Amplification kits. (Roche 454, Branford, Connecticut) The captured DNA library was emulsified and subjected to PCR in order to amplify the DNA template. The emulsion was chemically broken and the beads containing the DNA were recovered and up-regulated utilizing bead recovery reagents (Roche 454, Branford, Connecticut). The DNA library beads were loaded onto a PicoTiterPlate device and sequenced on the Genome Sequencer 454 Titanium instrument using the GS FLX titanium Sequencing Kit (Roche 454, Branford, Connecticut).

### Analytical processing of the reads, assembly and comparative analysis

cDNA sequence data for *C. oncophora* (egg, L1, L2, L3sh, L3ex, L4, adult males [M], and adult females [F]) and *O. ostertagi* (egg, L1, L2, L3sh, L3ex, L4, and adult) were screened for adaptor sequences using Seqclean http://compbio.dfci.harvard.edu/tgi/. The reads were then analyzed using the Newbler assembler v2.5 runMapping and those representing host contamination were removed from further consideration. The remaining reads were clustered using cd-hit-est [38] at 99% identity. The resulting representative reads were assembled into contigs using the Newbler assembler v2.5. Each stage was assembled individually and then the contigs were assembled by PHRAP (http://www.phrap.org/), using default settings, resulting in assembled transcripts. BLAT [39] was utilized (75% identity over 90% of length) to map the 8.7 million and the 11 million *C. oncophora* and *O. ostertagi* reads to the corresponding PHRAP assembly for expression profiling. The degree of fragmentation was determined as previously described [16].

Assembled transcripts were translated utilizing prot4est [40] and are available for acquisition and searching at http://nematode.net[15]. Predicted peptides were compared to the core eukaryotic genes (CEG’s) [41] using HMMER [42] to estimate the completeness of each transcriptome. Hits to the CEG’s were determined using the suggested cutoffs [41]. Predicted peptides were further analyzed using InterProScan [43] using tags to search for InterPro domains, GO terms, and Pfam domains. Putative secreted peptides were determined utilizing Phobius [44]. Peptides containing a signal peptide for secretion and no transmembrane sequences were designated as putatively secreted. Analyses of putatively secreted peptides were only performed on those shown to be up-regulated in at least one stage.

BLAST searches were used to compare the transcriptomes of *C. oncophora* and *O. ostertagi* to either genomic or transcriptomic data from thirteen other species subdivided into free-living nematodes (*Caenorhabditis elegans, C. japonica, C. remanei, C. brenneri, C. briggsae* and *Pristionchus pacificus*), Strongyloid parasites (*Dictyocaulus viviparus, Teladorsagia circumcincta, Trichostrongylus colubriformis and Oesophagostomum dentatum*) and non-Strongyloid nematode parasites (*A. suum*, *Trichinella spiralis*, and *B. malayi*) (1e-05, bit score of 50, and only the best hit). The BLAST output files are available at nematode.net [15]. Additional searches and comparisons were performed against the KEGG database (1e-05), and against each other (1e-05 and bit score of 50).

After reads were re-aligned to the transcripts using BLAT, the depth of coverage of each contig was calculated by dividing the lengths of all reads contributing to a contig by the length of the contig (Additional files 19 and 20). The coverage of a specific contig was then compared between the various stages using a binomial distribution and a p-value of 0.01 to determine the enrichment or depletion of reads. The hypergeometric function identifies nearly identical contig lists as EdgeR [45], but is much more lenient in significance cutoffs, resulting in more transcripts being identified as differentially-expressed (summary of the comparison is provided in Additional file 21 and the list of all differentially-expressed transcripts using each of the 2 methods is provided as Additional file 22). The up-regulated reads were grouped depending on whether they came from a free-living stage (egg-L3sh) or a parasitic stage (L3ex-adult). Prevalence of InterPro domains [46], GO categories [47], Pfam domains [48], KEGG [49] categories, and RNAi phenotypes was compared between the free-living and parasitic stages utilizing a G-test (*p* > 0.05).

Putative RNAi phenotypes were determined by comparing sequences derived herein to known *C. elegans* RNAi phenotypes as listed on WormMart [50]. In order to compare the *C. elegans* RNAi phenotypes to the free-living and parasitic stages of the nematodes in this study, the proteins in *C. elegans* were subdivided into two groups; all stages from the egg to the L3 dauer were considered akin to the “free-living” stages while dauer exit to adult worms were equated to the “parasitic” stages. If a polypeptide had multiple phenotypes, only the most severe was utilized in order of decreasing lethality i.e., embryonic lethal > larval lethal > sterile, growth > embryonic non-lethal > other. Identification of significant differences in categorical RNAi phenotype numbers between *C. elegans* and either *C. oncophora* or *O. ostertagi* was performed using a G-test (*p* > .05).

## Abbreviations

L2: Second stage larvae; L3: Third stage larvae; L3sh: L3 sheathed; L3ex: L3 exsheathed; F: Female; M: Male; A: Adult; cDNA: Complementary DNA; GO: Gene ontology; KEGG: Kyoto encyclopedia of genes and genomes; KO: KEGG ontology

## Competing interests

The authors declare that they have no competing interests.

## Authors’ contributions

MM, DSZ, PG, RBG conceived and designed the experiments. DSZ and JD provided the worms/RNA. EH, XG, and BR carried out experiments and analyses. EH, DSZ, RBG, PG and MM interpreted results and prepared the manuscript. All authors have read and approved the final manuscript.

## Supplementary Material

Additional file 1 ***C. oncophora *and *O. ostertagi *transcripts homologous to non-Strongylida only.** Description: The BLAST hits of the *C. oncophora* and *O. ostertagi* transcripts that share homology only to the non-Strongylida database. Click here for file

Additional file 2: Figure S1 Length distribution of peptides with and without homologues in other species. Description: Histogram of the length of peptides that have homologues in other species and those that do not have homologues i.e. are unique to either *C. oncophora* or *O. ostertagi.* Click here for file

Additional file 3 **Constitutively-expressed transcripts in *C. oncophora *and *O. ostertagi.*** Description: List of all transcripts constitutively-expressed in *C. oncophora* and *O. ostertagi.* Click here for file

Additional file 4 **KEGG categorization of constitutively-expressed transcripts in *C. oncophora.*** Description: Details of the KEGG matches for all constitutively-expressed transcripts in *C. oncophora.* Click here for file

Additional file 5 **KEGG categorization of constitutively-expressed transcripts in *O. ostertagi.*** Description: Details of the KEGG matches for all constitutively-expressed transcripts in *O. ostertagi.* Click here for file

Additional file 6 **InterPro annotation of *C. oncophora *peptides.** Description: Annotation based upon InterPro of all *C. oncophora* peptides. Click here for file

Additional file 7 **InterPro annotation of *O. ostertagi *peptides.** Description: Annotation based upon InterPro of all *O. ostertagi* peptides. Click here for file

Additional file 8 **Number of transcripts unique to a stage as well as the number up-regulated or down-regulated in a stage.** Description: Number of transcripts uniquely-expressed in a stage or differentially-expressed among stages. Click here for file

Additional file 9 **Prevalence of Pfam domains in *C. oncophora *(up-regulated transcripts only).** Description: The number of transcripts in each stage identified to hit a specific Pfam domain. Click here for file

Additional file 10 **Prevalence of Pfam domains in *O. ostertagi *(up-regulated transcripts only).** Description: The number of transcripts in each stage identified to hit a specific Pfam domain. Click here for file

Additional file 11 **Prevalence of GO biological processes in *C. oncophora *(up-regulated transcripts only).** Description: The number of transcripts in each stage associated with a specific GO biological process. Click here for file

Additional file 12 **Prevalence of GO cellular components in *C. oncophora *(up-regulated transcripts only).** Description: The number of transcripts in each stage associated with a specific GO Cellular component. Click here for file

Additional file 13 **Prevalence of GO molecular function in *C. oncophora *(up-regulated transcripts only).** Description: The number of transcripts in each stage associated with a specific GO Molecular function. Click here for file

Additional file 14 **Prevalence of GO biological processes in *O. ostertagi *(up-regulated transcripts only).** Description: The number of transcripts in each stage associated with a specific GO biological process. Click here for file

Additional file 15 **Prevalence of GO cellular components in *O. ostertagi *(up-regulated transcripts only).** Description: The number of transcripts in each stage associated with a specific GO Cellular component. Click here for file

Additional file 16 **Prevalence of GO molecular function in *O. ostertagi *(up-regulated transcripts only).** Description: The number of transcripts in each stage associated with a specific GO Molecular function. Click here for file

Additional file 17 **Prevalence of Intepro domains in *C. oncophora *(up-regulated transcripts only).** Description: The number of transcripts in each stage identified as a specific InterPro domain. Click here for file

Additional file 18 **Prevalence of Intepro domains in *O. ostertagi *(up-regulated transcripts only).** Description: The number of transcripts in each stage associated with a specific InterPro domain. Click here for file

Additional file 19 **Depth of coverage of *C. oncophora *transcripts.** Description: The calculated depth of coverage for all transcripts in *C. oncophora.* Click here for file

Additional file 20 **Depth of coverage of *O. ostertagi* transcripts.** Description: The calculated depth of coverage for all transcripts in *O. ostertagi.* Click here for file

Additional file 21 **Summary of comparison of differentially-expressed transcripts based on hypergeometric binomial distribution and EdgeR.** Description: The number of transcripts identified as being differentially-expressed using the two different methods. Click here for file

Additional file 22 **Differentially-expressed transcripts in *C. oncophora *and *O. ostertagi *using hypergenometric and EdgeR analysis.** Description: List of all identified differentially-expressed transcripts in *C. oncophora* and *O. ostertagi* using hypergenometric and EdgeR analysis. Click here for file

## References

[B1] Blaxter ML De Ley P Garey JR Liu LX Scheldeman P Vierstraete A Vanfleteren JR Mackey LY Dorris M Frisse LM A molecular evolutionary framework for the phylum Nematoda Nature 1998 392 6671 71 75 10.1038/32160 9510248

[B2] Wolstenholme AJ Fairweather I Prichard R Von Samson-Himmelstjerna G Sangster NC Drug resistance in veterinary helminths Trends Parasitol 2004 20 10 469 476 10.1016/j.pt.2004.07.010 15363440

[B3] Claerebout E Hilderson H Meeus P De Marez T Behnke J Huntley J Vercruysse J The effect of truncated infections with Ostertagia ostertagi on the development of acquired resistance in calves Vet Parasitol 1996 66 3–4 225 239 9017885 10.1016/s0304-4017(96)01012-6

[B4] Smith HJ Archibald RM The effects of age and previous infection on the development of gastrointestinal parasitism in cattle Can J Comparative Med 1968 32 4 511 517 PMC1319290 4234780

[B5] Abubucker S Zarlenga DS Martin J Yin Y Wang Z McCarter JP Gasbarree L Wilson RK Mitreva M The transcriptomes of the cattle parasitic nematode Ostertagia ostartagi Vet Parasitol 2009 162 1–2 89 99 19346077 10.1016/j.vetpar.2009.02.023 PMC2677129

[B6] Cantacessi C Mitreva M Campbell BE Hall RS Young ND Jex AR Ranganathan S Gasser RB First transcriptomic analysis of the economically important parasitic nematode, Trichostrongylus colubriformis, using a next-generation sequencing approach Infect Genet Evol 2010 10 8 1199 1207 10.1016/j.meegid.2010.07.024 20692378 PMC3666958

[B7] Parkinson J Mitreva M Whitton C Thomson M Daub J Martin J Schmid R Hall N Barrell B Waterston RH A transcriptomic analysis of the phylum Nematoda Nat Genet 2004 36 12 1259 1267 10.1038/ng1472 15543149

[B8] Cantacessi C Mitreva M Jex AR Young ND Campbell BE Hall RS Doyle MA Ralph SA Rabelo EM Ranganathan S Massively parallel sequencing and analysis of the Necator americanus transcriptome PLoS Negl Trop Dis 2010 4 5 e684 10.1371/journal.pntd.0000684 20485481 PMC2867931

[B9] Wang Z Abubucker S Martin J Wilson RK Hawdon J Mitreva M Characterizing Ancylostoma caninum transcriptome and exploring nematode parasitic adaptation BMC Genomics 2010 11 307 10.1186/1471-2164-11-307 20470405 PMC2882930

[B10] Yin Y Martin J McCarter JP Clifton SW Wilson RK Mitreva M Identification and analysis of genes expressed in the adult filarial parasitic nematode Dirofilaria immitis Int J Parasitol 2006 36 7 829 839 10.1016/j.ijpara.2006.03.002 16697384

[B11] Nisbet AJ Cottee PA Gasser RB Genomics of reproduction in nematodes: prospects for parasite intervention? Trends Parasitol 2008 24 2 89 95 10.1016/j.pt.2007.12.001 18182326

[B12] Wang Z Martin J Abubucker S Yin Y Gasser RB Mitreva M Systematic analysis of insertions and deletions specific to nematode proteins and their proposed functional and evolutionary relevance BMC Evol Biol 2009 9 23 10.1186/1471-2148-9-23 19175938 PMC2644674

[B13] Mitreva M Zarlenga DS McCarter JP Jasmer DP Parasitic nematodes - from genomes to control Vet Parasitol 2007 148 1 31 42 10.1016/j.vetpar.2007.05.008 17560034

[B14] Parra G Bradnam K Korf I CEGMA: a pipeline to accurately annotate core genes in eukaryotic genomes Bioinformatics 2007 23 9 1061 1067 10.1093/bioinformatics/btm071 17332020

[B15] Martin J Abubucker S Heizer E Taylor CM Mitreva M Nematode.net update 2011: addition of data sets and tools featuring next-generation sequencing data Nucleic Acids Res 2012 40 Database issue D720 D728 22139919 10.1093/nar/gkr1194 PMC3245159

[B16] Mitreva M McCarter JP Martin J Dante M Wylie T Chiapelli B Pape D Clifton SW Nutman TB Waterston RH Comparative genomics of gene expression in the parasitic and free-living nematodes Strongyloides stercoralis and Caenorhabditis elegans Genome Res 2004 14 2 209 220 10.1101/gr.1524804 14762059 PMC327096

[B17] Mitreva M McCarter JP Arasu P Hawdon J Martin J Dante M Wylie T Xu J Stajich JE Kapulkin W Investigating hookworm genomes by comparative analysis of two Ancylostoma species BMC Genomics 2005 6 58 10.1186/1471-2164-6-58 15854223 PMC1112591

[B18] Burglin TR Lobos E Blaxter ML Caenorhabditis elegans as a model for parasitic nematodes Int J Parasitol 1998 28 3 395 411 10.1016/S0020-7519(97)00208-7 9559358

[B19] Couteau F Guerry F Muller F Palladino F A heterochromatin protein 1 homologue in Caenorhabditis elegans acts in germline and vulval development EMBO Rep 2002 3 3 235 241 10.1093/embo-reports/kvf051 11850401 PMC1084015

[B20] McDermott SR Noor MA The role of meiotic drive in hybrid male sterility Philos Trans R Soc Lond B Biol Sci 2010 365 1544 1265 1272 10.1098/rstb.2009.0264 20308102 PMC2871811

[B21] Brodin TN Heath S Sacks DL Genes selectively expressed in the infectious (metacyclic) stage of Leishmania major promastigotes encode a potential basic-zipper structural motif Mol Biochem Parasitol 1992 52 2 241 250 10.1016/0166-6851(92)90056-P 1620162

[B22] Chazin WJ Relating form and function of EF-hand calcium binding proteins Acc Chem Res 2011 44 3 171 179 10.1021/ar100110d 21314091 PMC3059389

[B23] Don TA Oksov Y Lustigman S Loukas A Saposin-like proteins from the intestine of the blood-feeding hookworm, Ancylostoma caninum Parasitology 2007 134 Pt 3 427 436 17109779 10.1017/S003118200600148X

[B24] Goyal K Pinelli C Maslen SL Rastogi RK Stephens E Tunnacliffe A Dehydration-regulated processing of late embryogenesis abundant protein in a desiccation-tolerant nematode FEBS Lett 2005 579 19 4093 4098 10.1016/j.febslet.2005.06.036 16023104

[B25] Rougvie AE Ambros V The heterochronic gene lin-29 encodes a zinc finger protein that controls a terminal differentiation event in Caenorhabditis elegans Development 1995 121 8 2491 2500 7671813 10.1242/dev.121.8.2491

[B26] Daub J Loukas A Pritchard DI Blaxter M A survey of genes expressed in adults of the human hookworm, Necator americanus Parasitology 2000 120 Pt 2 171 184 10726278 10.1017/s0031182099005375

[B27] Ito N Mita M Takahashi Y Matsushima A Watanabe YG Hirano S Odani S Novel cysteine-rich secretory protein in the buccal gland secretion of the parasitic lamprey, Lethenteron japonicum Biochem Biophys Res Commun 2007 358 1 35 40 10.1016/j.bbrc.2007.04.065 17467660

[B28] Hopla CE Durden LA Keirans JE Ectoparasites and classification Rev Sci Tech 1994 13 4 985 1017 7711316 10.20506/rst.13.4.815

[B29] Cantacessi C Campbell BE Visser A Geldhof P Nolan MJ Nisbet AJ Matthews JB Loukas A Hofmann A Otranto D A portrait of the “SCP/TAPS” proteins of eukaryotes–developing a framework for fundamental research and biotechnological outcomes Biotechnol Adv 2009 27 4 376 388 10.1016/j.biotechadv.2009.02.005 19239923

[B30] Hawdon JM Narasimhan S Hotez PJ Ancylostoma secreted protein 2: cloning and characterization of a second member of a family of nematode secreted proteins from Ancylostoma caninum Mol Biochem Parasitol 1999 99 2 149 165 10.1016/S0166-6851(99)00011-0 10340481

[B31] Moreno Y Gros PP Tam M Segura M Valanparambil R Geary TG Stevenson MM Proteomic analysis of excretory-secretory products of Heligmosomoides polygyrus assessed with next-generation sequencing transcriptomic information PLoS Negl Trop Dis 2011 5 10 e1370 10.1371/journal.pntd.0001370 22039562 PMC3201918

[B32] Larance M Bailly AP Pourkarimi E Hay RT Buchanan G Coulthurst S Xirodimas DP Gartner A Lamond AI Stable-isotope labeling with amino acids in nematodes Nat Methods 2011 8 10 849 851 10.1038/nmeth.1679 21874007 PMC3184259

[B33] Loukas A Maizels RM Helminth C-type lectins and host-parasite interactions Parasitol Today 2000 16 8 333 339 10.1016/S0169-4758(00)01704-X 10900481

[B34] Elling AA Mitreva M Recknor J Gai X Martin J Maier TR McDermott JP Hewezi T Mc KBD Davis EL Divergent evolution of arrested development in the dauer stage of Caenorhabditis elegans and the infective stage of Heterodera glycines Genome Biol 2007 8 10 R211 10.1186/gb-2007-8-10-r211 17919324 PMC2246285

[B35] Balachandar R Lu NC Nutritional requirements for pantothenate, pantethine or coenzyme A in the free-living nematode Caenorhabditis elegans Nematology 2005 7 761 766 10.1163/156854105775142900

[B36] Kaplan RM Drug resistance in nematodes of veterinary importance: a status report Trends Parasitol 2004 20 10 477 481 10.1016/j.pt.2004.08.001 15363441

[B37] Snider TG 3rd Ochoa R Williams JC Menetrier’s disease. Pre-Type II and Type II ostertagiosis in cattle Am J Pathol 1983 113 3 410 412 6650666 PMC1916346

[B38] Li W Godzik A Cd-hit: a fast program for clustering and comparing large sets of protein or nucleotide sequences Bioinformatics 2006 22 13 1658 1659 10.1093/bioinformatics/btl158 16731699

[B39] Kent WJ BLAT–the BLAST-like alignment tool Genome Res 2002 12 4 656 664 11932250 10.1101/gr.229202 PMC187518

[B40] Wasmuth JD Blaxter ML Prot4EST: Translating Expressed Sequence Tags from neglected genomes BMC Bioinformatics 2004 5 187 10.1186/1471-2105-5-187 15571632 PMC543579

[B41] Parra G Bradnam K Ning Z Keane T Korf I Assessing the gene space in draft genomes Nucleic Acids Res 2009 37 1 289 297 10.1093/nar/gkn916 19042974 PMC2615622

[B42] Eddy SR Profile hidden Markov models Bioinformatics 1998 14 9 755 763 10.1093/bioinformatics/14.9.755 9918945

[B43] Quevillon E Silventoinen V Pillai S Harte N Mulder N Apweiler R Lopez R InterProScan: protein domains identifier Nucleic Acids Res 2005 33 W116 W120 10.1093/nar/gki442 15980438 PMC1160203

[B44] Kall L Krogh A Sonnhammer EL A combined transmembrane topology and signal peptide prediction method J Mol Biol 2004 338 5 1027 1036 10.1016/j.jmb.2004.03.016 15111065

[B45] Robinson MD McCarthy DJ Smyth GK edgeR: a Bioconductor package for differential expression analysis of digital gene expression data Bioinformatics 2010 26 1 139 140 10.1093/bioinformatics/btp616 19910308 PMC2796818

[B46] Hunter S Jones P Mitchell A Apweiler R Attwood TK Bateman A Bernard T Binns D Bork P Burge S InterPro in 2011: new developments in the family and domain prediction database Nucleic Acids Res 2012 40 Database issue D306 D312 22096229 10.1093/nar/gkr948 PMC3245097

[B47] The Gene Ontology Consortium Gene Ontology Annotations and Resources Nucleic Acids Res 2013 41 (Database issue) D530 D535 23161678 10.1093/nar/gks1050 PMC3531070

[B48] Punta M Coggill PC Eberhardt RY Mistry J Tate J Boursnell C Pang N Forslund K Ceric G Clements J The Pfam protein families database Nucleic Acids Res 2012 40 Database issue D290 D301 22127870 10.1093/nar/gkr1065 PMC3245129

[B49] Okuda S Yamada T Hamajima M Itoh M Katayama T Bork P Goto S Kanehisa M KEGG Atlas mapping for global analysis of metabolic pathways Nucleic Acids Res 2008 36 Web Server issue W423 W426 18477636 10.1093/nar/gkn282 PMC2447737

[B50] Schwarz EM Antoshechkin I Bastiani C Bieri T Blasiar D Canaran P Chan J Chen N Chen WJ Davis P WormBase: better software, richer content Nucleic Acids Res 2006 34 Database issue D475 D478 16381915 10.1093/nar/gkj061 PMC1347424

