# Supplementary figures and images for: Transcriptome analyses reveal protein and domain families that delineate stage-related development in the economically important parasitic nematodes, Ostertagia ostertagi and Cooperia oncophora
Source: BMC Genomics. 2013 Feb 22;14:118. doi: 10.1186/1471-2164-14-118 (PMC3599158; doi:10.1186/1471-2164-14-118)

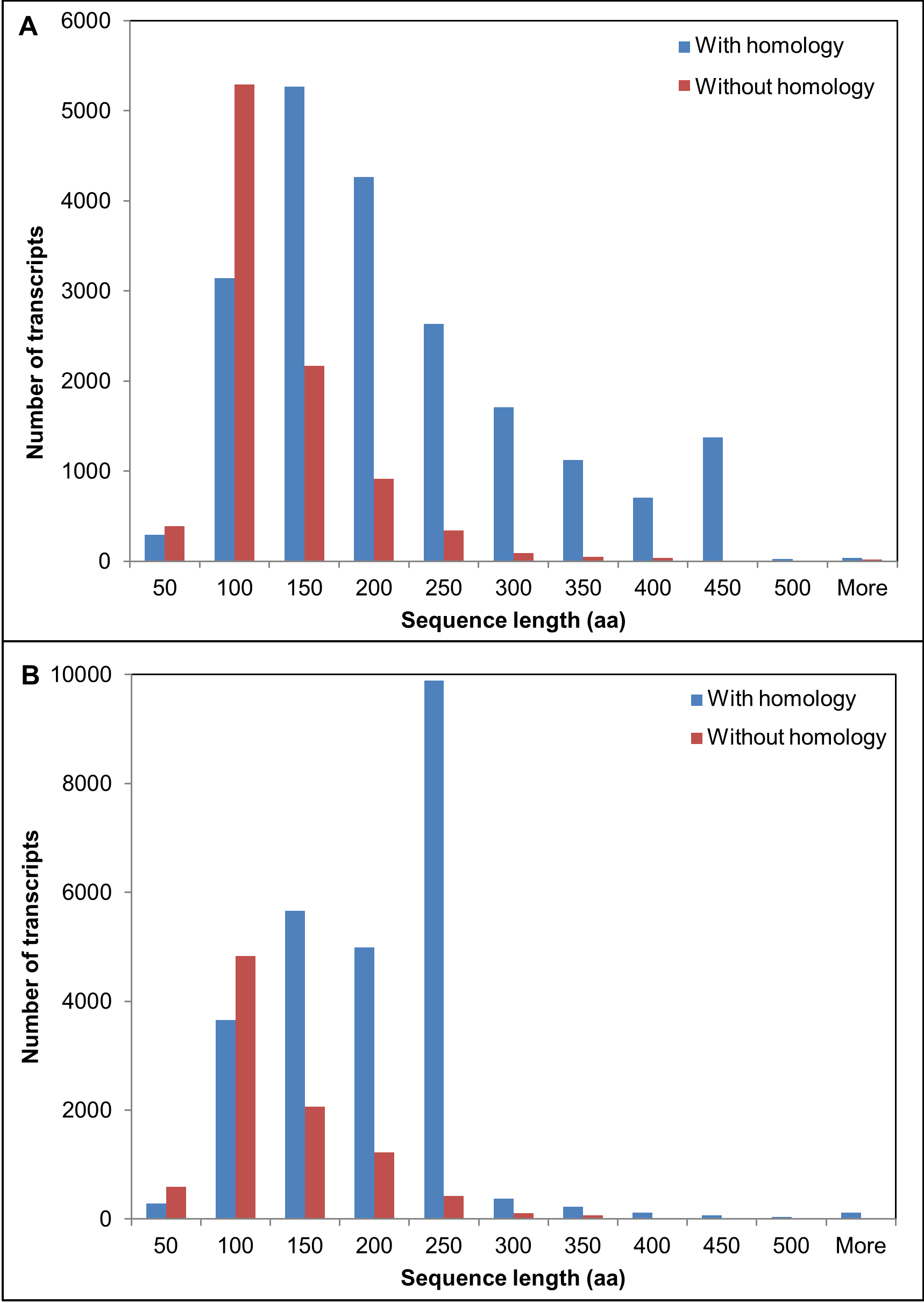

Supplement: Additional file 2: Figure S1 — Length distribution of peptides with and without homologues in other species. Description: Histogram of the length of peptides that have homologues in other species and those that do not have homologues i.e. are unique to either C. oncophora or O. ostertagi. [file 1471-2164-14-118-S2.tiff]
